# Modern Strategies for Heterocycle Synthesis

**DOI:** 10.3390/molecules25112476

**Published:** 2020-05-27

**Authors:** Gianfranco Favi

**Affiliations:** Department of Biomolecular Sciences, Section of Chemistry and Pharmaceutical Technologies, University of Urbino “Carlo Bo”, Via I Maggetti 24, 61029 Urbino (PU), Italy; gianfranco.favi@uniurb.it

Heterocycles constitute the largest and most diverse family of organic compounds that have received extensive interest owing to their popularity in many natural products, pharmaceuticals, and materials. It is estimated that of the over 50 million registered organic compounds in Chemical Abstracts [1], more than half are heterocycles, and the number is still increasing. Most frequently, nitrogen and oxygen heterocycles, or various positional combinations of nitrogen atoms, oxygen, and sulfur in five or six-membered rings, can be found. Due to the central role of heterocycles in chemistry and biology, new advances in synthetic methodologies that allow rapid access to a wide variety of functionalized heterocyclic compounds are of critical importance to the chemical community. For these reasons, a Special Issue collecting research on some aspects of innovative strategies for assembling heterocycles represents a good opportunity for dissemination of recent progress in this field. The vastness of this topic is documented by four review articles.

In their review, Stodulski and co-workers [2] highlight recent progress in the application of metal-free, visible-light-mediated catalysis for assembling five- and six-member heterocyclic scaffolds containing nitrogen and oxygen heteroatoms, particularly focused on the use of inexpensive organic dyes as an excellent alternative to the typical transition-metal complexes. García-Tellado’s group [3] presents the nature of the different physicochemical factors affecting the valence isomerism between 2*H*-pyrans (2HPs) and 1-oxatrienes, and describes the most versatile synthetic methods reported in recent literature to access 2HPs. In more detail, a selection of the most transited routes able to generate these rings with a convenient amount of structural diversity, including the proper Knoevenagel reaction, the tandem propargyl Claisen rearrangement/*H*-shift reactions hosted by propargyl vinyl ethers [1,3], the cycloisomerization of diynes, and the Stille coupling of vinyl iodides and vinyl stannanes, is reported. The contribution of Lee and his group [4] provides an overview of the biological roles and synthetic strategies of saturated cyclic ethers, covering some of the most studied, and newly discovered, related natural products in recent years. This review also reports several promising and newly developed synthetic methods, emphasizing 3–7 membered rings. Chiummiento and co-workers [5] contribute to this Special Issue with a review describing recent developments in benzofurans synthesis. More specifically, new intramolecular and intermolecular C–C and/or C–O bond-forming processes, with transition-metal catalysis or metal-free are summarized.

In this Special Issue, Liu, Zhao and co-authors [6] report the first example of the generation of an indole/thiophene/pyrrole/pyridine/naphthalene/benzene-fused *N*-heterocycle library through an AuPPh_3_Cl/AgSbF_6_-catalyzed cascade reaction between amine nucleophiles and alkynoic acids in water. Low catalyst loading, good to excellent yields, high efficiency in bond formation (three new bonds together with two rings), excellent selectivity, great tolerance of functional groups, and extraordinarily broad substrate scope are features of this green cascade process.

The advantages of solid-phase synthesis in time-efficient and traceless preparation of ketones via acid-labile enol ethers are described in the article by Krchňák and co-authors [7]. The practicality of this synthetic strategy on the solid-phase construction of pyrrolidine-2,4-diones, which represent the core structure of several natural products, including tetramic acid, is also demonstrated. 

A series of chromeno[3,4-c]chromen-6-one derivatives, a new type of dihydrocoumarins, are synthesized by Xu, Yang et al. [8] via Michael addition, transesterification and nucleophilic addition from the reaction of 3-trifluoroacetyl coumarins and phenols in the presence of an organic base. For these compounds, the in vitro antifungal activity is assessed against two fungal strains with the mycelial growth rate method.

In this Issue, a couple of articles concerning the copper(I)-catalyzed azide-alkyne cycloaddition (CuAAC) (the “click reaction”) are presented. In this regard, Bálint and co-workers [9] describe a facile and efficient method for the synthesis of new (1,2,3-triazol-4-yl)methyl phosphinates/phosphates by the Cu(I)-catalyzed 1,3-dipolar (Huisgen) cycloaddition of organic azides and prop-2-ynyl phosphinate/phosphate. In the other article, Balova’s group [10] first reports the synthesis of 6-aryl-4-azidocinnolines through the Richter-type cyclization of 2-ethynyl-4-aryltriazenes with the formation of 4-bromo-6-arylcinnolines and nucleophilic substitution of a bromine atom with an azide functional group, then their use in both CuAAC with terminal alkynes and SPAAC with diazacyclononyne, yielding 4-triazolylcinnolines.

This Special Issue presents three contributions of multicomponent reactions (MCRs), in which access to different molecular skeletons is realized. Participation of various alcohols in the construction of coumarin-fused pyrazolo[3,4-*b*]pyridines via a silica sulfuric acid (SSA)-catalyzed three-component domino reaction under microwave irradiation is presented for the first time by Lin, Wang and co-authors [11]. In another work, the group of Palacios and Vicario [12] reports a phosphoric acid-catalyzed MCR procedure for the synthesis of highly functionalized γ-lactam derivatives by the reaction of benzaldehyde, amines and acetylenedicarboxylate. Another article dealing with MCR strategy is described by our group [13]. Combining sequential azidation, Staudinger, and aza-Wittig reactions with CS_2_ on α-halohydrazones in a one-pot protocol, variously substituted 1-amino-1*H*-imidazole-2(3*H*)-thiones are directly accessible in good yields and with complete control of regioselectivity. In addition, the concurrent presence of reactive appendages on the obtained scaffolds also ensures post-modifications toward *N*-bridgeheaded heterobicyclic structures.

Although the synthesis of terpyridines by one-pot reaction of acetylpyridines, aromatic aldehydes and ammonia is presented in the literature as an infallible synthetic method, there is ample precedent for the formation of a variety of alternative products. Based on this assumption, Constable et al. [14] provides another example of an unexpected product and a systematic survey of the products of such reactions.

Utilizing a pharmacophore hybridization approach, a novel series of 28 new heterobivalent β-carbolines bearing an acylhydrazone bond is reported by Zhang and co-workers [15]. The results of their in vitro antiproliferative activity using the MTT-based assay against five cancer cell lines (LLC, BGC-823, CT-26, Bel-7402, and MCF-7) contribute to the further elucidation of the biological regulatory role of these compounds, as well as providing helpful information on the development of vascular targeting antitumor drugs.

In this Issue, there are two articles describing the synthesis of heterocycles and their functionalization using metal-free approaches. In one of them, Trofimov and Nenajdenko’s team [16] elaborates an efficient pathway towards trifluoromethylated oxazinopyridines on the base of a one-pot, metal-free 1:2 assembly of pyridines and CF_3_-ynone. Target heterocycles are prepared in a stereoselective manner and up to quantitative yields. In the other work, through the direct arylation of halopurines with aromatic compounds, facilitated by the combination of triflic acid and fluoroalcohol, various aryl-substituted purine derivatives are synthesized by the Takenaga and Dohi group [17]. This metal-free method is complementary to conventional coupling reactions using metal catalysts and reagents for the syntheses of aryl-substituted purine analogues.

A publication on a TMSBr-promoted cascade cyclization of *ortho*-propynol phenyl azides for the synthesis of 4-bromo quinolines is reported by Xiao and his group [18]. Moreover, a variety of functionalized compounds with molecular diversity at C4 position of quinolines are obtained through the subsequent coupling or nucleophilic reactions. 

Finally, a contribution of Tomassetti, Marcantoni et al. [19] deals with the synthesis of polysubstituted 5-acylamino-1,3-thiazoles via a Hantzsch heterocyclization reaction of α-chloroglycinates with thiobenzamides or thioureas. As result, the pharmaceutically relevant target compounds are obtained under mild conditions from readily available, inexpensive building blocks through an environmentally benign process that requires no stringent control of reaction parameters/atmosphere and no catalysts.

As guest editor, I hope that you find this Special Issue highlighting contributions in the vast field of heterocyclic chemistry both informative and inspiring.
